# Considering planetary environments in origin of life studies

**DOI:** 10.1038/s41467-018-07493-3

**Published:** 2018-12-12

**Authors:** Laura M. Barge

**Affiliations:** NASA Jet Propulsion Laboratory, California Institute of Technology, 4800 Oak Grove Drive, Pasadena, CA 91109 USA

## Abstract

Early Earth geological conditions would have affected prebiotic chemistry: particularly the lack of atmospheric oxygen, presence of dissolved iron, and increased high-energy radiation. Incorporating planetary conditions into origin-of-life studies can also advance our search for life on other worlds.

## Introduction

We only have one example of life in the Universe thus far, and it is still hotly debated which chemical reactions, and underlying conditions, led to the origin of Earth’s biosphere. Origin of life chemistry experiments often aim to recapitulate reactions or molecules/molecular processes that resemble species that were present in the last universal common ancestor (LUCA) or that could have eventually led to LUCA. However, considerations for such reactions need to be founded in what we know of early Earth environments; the suite of reactions that were most likely to have proceeded under early Earth conditions is only a subset of chemistries that are physically possible in the laboratory. Major factors that would have affected prebiotic chemistry—and should be considerations in designing prebiotic experiments—include the lack of atmospheric O_2_, the presence of dissolved ferrous iron and the resulting reactive minerals, and the effects of photochemistry driven by increased surface/atmospheric radiation. As we search for evidence of life on other worlds, it becomes increasingly important to understand the broad types of planetary conditions that can lead to the emergence of life, so that we can recognize them elsewhere in the solar system. Particularly it is important to not make assumptions about the specific conditions (able to be simulated in the laboratory) that a type of environment does or does not contain, or what list of environments may have a particular desired condition, since there is great unexpected variety in geological and planetary systems both at the macro and micro scale.

### Anoxic conditions

One of the most profound geochemical changes in Earth’s history was the rise of atmospheric oxygen due to biological photosynthesis^1^. Early Earth’s atmosphere was predominantly thought to be rich in N_2_/CO_2_/H_2_O/SO_2_ and thus the oceans would have been mildly acidic and anoxic^2–4^, although there could have been some oxidizing conditions in a stratified ocean^5^. This would have allowed for relatively high concentrations of dissolved ferrous iron (Fe^2+^) as well as precipitation of reactive, mixed valence iron minerals in the water column and hydrothermal mixing zones^6^. Fe^2+^ is a reactive ion that can drive various prebiotically relevant reactions such as Fenton chemistry and can also serve as a cofactor for RNA catalysis^7^. Iron minerals also provide highly reactive surfaces for redox chemistry of inorganic and organic compounds and are involved in elemental cycling; other minerals (including clays, other metal oxides, zeolites) and silica gels or colloids^8^ can also be effective in concentrating reactants and products even in an aqueous system, including nucleobases and amino acids.

Often the default for lab experiments is to do them under ambient atmosphere, mostly due to the practical difficulties of maintaining rigorously oxygen-free environments. Even trace amounts of oxygen (low ppm concentrations) can change an experimental result significantly. For origin of life laboratory simulations, results in ambient conditions may be instructive but it is essential to also explore the chemistry when O_2_ is rigorously excluded from the system. In an early Earth context, one major consequence of anoxic conditions was the presence of dissolved Fe^2+^ ions, rather than these being immediately precipitated or oxidized. It is always a good idea to try supposed prebiotic reactions not only in anoxic environments, but also in the presence of dissolved ferrous iron at relevant concentrations. This necessitates techniques such as oxygen-free glove boxes, argon-purged vials, and freeze-pump-thaw degassing of water prior to its use in experiments. Analysis under anoxic conditions must be carefully planned as well, since even a few minutes of exposure to the atmosphere on the way to an instrument can significantly oxidize a sample (of, for example, reduced iron minerals), including oxygen diffusion into supposedly sealed sample containers when shipping, or even oxidation that occurs during analysis. Many interesting prebiotic redox reactions can occur driven by Fe(II)-containing minerals or in Fe^2+^-containing fluids, and so oxidation of iron can also occur chemically and not always be due to ambient atmosphere contamination. Proper controls and sample handling are essential for this type of work and it is recommended to verify iron oxidation state at various time points throughout an experiment (via colorimetry, electrochemistry, or other techniques) to distinguish between these oxidation mechanisms. The elevated atmospheric CO_2_ concentrations on early Earth relative to present day would have equilibrated with the early oceans, likely leading to a slightly acidic environment (pH ~5–7) which would affect reactions as well. It is also important to measure and control pH values in prebiotic chemistry experiments to ensure that reactions are being conducted at relevant conditions.

### Radiation and photochemistry

On the early Earth, high-energy solar radiation that reached the surface was higher than today, due to the increased rotation of young solar-type stars (which slows down with stellar age) and also the lack of UV-absorbing ozone in the atmosphere (which is produced from atmospheric oxygen). Thus, photochemistry as well as surface reactions involving UV radiation may have been significant. Many studies have explored the effects of photochemistry on origin of life relevant reactions, for example, the ability of photo-catalytic minerals to drive proto-metabolic cycles and/or organic synthesis (e.g.^9^). There are various factors that could protect environments from radiation, even on the surface or in shallow water, allowing reactions to proceed in the absence of these effects—for example, dissolved Fe^2+^ as well as some iron minerals are effective UV absorbers, though in these same environments UV-induced Fenton chemistry could also produce radicals that are harmful to organics and/or life^10,11^. Though many prebiotic reactive settings e.g. in the ocean or at the seafloor would not have had direct interaction with solar radiation, the effects of photochemistry can still be felt through the production of oxidants that might permeate into the water column and react with Fe^2+^ and other species. Regardless of whether experiments are directly simulating surface or atmospheric reactions, versus ocean or other environments where radiation is not as much of a factor, it is still important to thoroughly consider the effects of photochemistry on the geochemical system. A seafloor system could still be affected by the presence of oxidants produced photochemically in the atmosphere or waters above; for example, it has been suggested that nitrate/nitrite species—species that are highly reactive with dissolved iron and iron minerals—produced by photochemistry and/or lightning in the atmosphere could be important for the emergence of metabolism in a deep-sea vent setting^12^.

### Predicting conditions but not limiting possible environments

Depending on the reaction being studied, various conditions are considered favorable or unfavorable in terms of pH, temperature, condition cycling, presence of certain mineral catalysts, etc. Currently there is no one set of experimental conditions that is universally agreed on by the origin of life community to be capable of facilitating all the reactions deemed necessary for life to emerge. It is also not known whether the origin of life all happened in one geological setting or whether multiple different settings were involved (e.g.^13^.); it also may not matter as long as all the conditions for necessary reactions were met (e.g. in a single setting with gradients that may cycle through various conditions). For certain reactions there are multiple plausible geochemical scenarios, for example the synthesis of amino acids which can be accomplished via various mechanisms; for others, the geologically reasonable conditions may be more limited, such as the emergence of certain metabolic pathways. It is indeed important to make sure successful experimental conditions are fundamentally reasonable for early Earth. However it is also important to not let experimental results limit what types of environments we consider relevant for the origin of life, because we do not know the sum of what early Earth environments provided exactly which physical or chemical conditions. For example, it is proposed that thermal gradients in small pore spaces can drive nucleobase synthesis and favor formation of long oligomers;^14,15^ this expands the possibilities for nucleotide reactions in surface or underwater vent settings that host rock/mineral porous structures, but could also potentially occur in any environment that is found to host porous media with a thermal gradient. It is not necessary for origin of life laboratory experiments to produce end-to-end predictions of geological environments that lead to that process; the goal should instead be to explore origin of life possibilities in any planetary setting we may find that hosts the relevant conditions. This is especially true in the context of astrobiology and planetary exploration, where there have been many surprising discoveries involving chemical conditions and geological settings that we would not have predicted to exist, but are found in our solar system and may also provide possibilities for life to emerge on extrasolar planets.
